# Supplementary material for: The microbiome of a bacterivorous marine choanoflagellate contains a resource-demanding obligate bacterial associate
Source: Nat Microbiol. 2022 Aug 15;7(9):1466–79. doi: 10.1038/s41564-022-01174-0 (PMC9418006; doi:10.1038/s41564-022-01174-0)
Supplement: Supplementary file 1 — 1. Protologues for new Candidatus taxa identified from metagenomic analysis of Bicosta minor sorted single-cells. 2. Supplementary Figures 1 and 2 and corresponding legends. [file 41564_2022_1174_MOESM1_ESM.pdf]

nature portfolio

https://doi.org/10.1038/s41564-022-01174-0

Supplementary information

The microbiome of a bacterivorous marine choanoflagellate contains a resource-demanding obligate bacterial associate

In the format provided by the authors and unedited

Protologues for new *Candidatus* taxa identified from metagenomic analysis of *Bicosta minor* sorted single-cells.

Description of Candidatus Comchoanobacterales ord. nov.

Candidatus Comchoanobacterales *(Com.choa.no.bac.te.ra'les. N.L. masc. n. Comchoanobacter* type genus of the order; N.L. suff. *–ales* to denote an order; N.L. fem. pl. n. *Comchoanobacterales*, the *Comchoanobacter* order*).*

A bacterial order identified by metagenomic analyses from bulk seawater samples and single-cell sorted cells collected from various marine sites. This is the name for the alphanumeric GTDB order UBA7916[44]. This order has been assigned by GTDB working on GTDB Release 89[44,77] and by the phylogenomic tree displayed in this study (Extended Fata Fig. 7) to the class Gammaproteobacteria.

Description of *Candidatus* Comchoanobacteraceae fam. nov.

Candidatus Comchoanobacteraceae (Com.choa.no.bac.te.ra.ce'ae. N.L. masc. n. *Comchoanobacter* type genus of the family; N.L. suff. *–aceae* to denote a family; N.L. fem. pl. n. *Comchoanobacteraceae,* the *Comchoanobacter* family).

A bacterial family identified by metagenomic analyses (bulk seawater sampling and single-cell sorting). This is a name for the alphanumeric GTDB family UBA1515[44,77]. The family is assigned to the order Comchoanobacterales.

Description of *Candidatus* Comchoanobacter gen. nov.

Candidatus Comchoanobacter (Com.choa.no.bac'ter. L. pref. *Cum-*, with; Gr. fem. n. *khoánē,* funnel; N.L. masc. n. *bacter*, staff; N.L. masc. n. *Comchoanobacter* a bacterium associated with a choanoflagellate).

A bacterial genus identified by single-cell sorted metagenomic analyses. The genus includes all bacteria with genomes that show ≥60% average amino acid identity (AAI) to the type genome from the type species *Candidatus* Comchoanobacter bicosticola. This genus is assigned to the order Comchoanobacterales and to the family Comchoanobacteraceae.

Description of *Candidatus* Comchoanobacter bicosticola sp. nov.

Candidatus Comchoanobacter bicosticola (bi.cos.ti'co.la. N.L. fem. n. *bicosta* the choanoflagellate genus *Bicosta*; L. suff. *-cola* inhabitant of; N.L. n. *bicosticola* an inhabitant of *Bicosta*).

A bacterial species identified by single-cell sorted metagenomic analyses. This species includes all bacteria with genomes that show ≥95% average nucleotide identity (ANI) to the type genome, which has been assigned the SAG ID Comchoano-1 and which is available via NCBI accession CP092900. The Comchoano-1 16S rRNA gene sequence is available via NCBI accession OM801198. The relative evolutionary distance (RED) is 0.83 to the closest relatives in GTDB release 95, as well as after re-calculation of all Comchoanobacterales identified in the present study. The phylogenetic position within the Comchoanobacterales order and the Comchoanobacteraceae family has been analyzed using both the reference genome (Extended Data Fig. 7b) and the 16S rRNA gene sequence (Fig. 2a). The GC content of the type genome is 39.3% and the genome length is 1.01 Mbp. The genome is high-quality, in a single circular chromosome, with standard completion estimates (based on presence of single copy marker genes of Bacteria[17]) suggesting 96.55% completion and 0% contamination. However, this is likely an underestimate of the genome completion due to the single circular chromosome sequence for the genome, as well as the fact that no additional novelty was detected across multiple single cells. The type genome *Candidatus* Comchoanobacter bicosticola sp. nov. originated from the North Pacific Ocean, from 20 m water depth. The genomes have limitations metabolically, apparently unable to synthesis fatty acids, vitamins, amino acids, or perform glycolysis, while encoding for a Type IV secretion system, pentose phosphate pathway, oxidative phosphorylation, a rhodopsin, and a putative ATP/ADP translocase.

Description of *Candidatus* Synchoanobacter gen. nov.

Candidatus Synchoanobacter (Syn.choa.no.bac'ter. Gr. pref. *Sún-*, with; Gr. fem. n. *khoánē,* funnel; N.L. masc. n. *bacter*, staff; N.L. masc. n. *Synchoanobacter* a bacterium associated with a choanoflagellate).

A bacterial genus identified by single-cell sorted metagenomic analyses. The genus includes all bacteria with genomes that show ≥60% average amino acid identity (AAI) to the type genome from the type species *Candidatus* Synchoanobacter obligatus. This genus is assigned to the order Comchoanobacterales and to the family Comchoanobacteraceae.

Description of *Candidatus* Synchoanobacter obligatus sp. nov.

Candidatus Synchoanobacter obligatus (o.bli.ga'tus. L. pass. part. nom. n. *obligatus* bound by obligation).

A bacterial species identified by metagenomic analyses. This species includes all bacteria with genomes that show ≥95% average nucleotide identity (ANI) to the type genome, which has been assigned the SAG ID Comchoano-2 and which is available via NCBI Accession JAKUDN000000000 (The version described in this paper is version JAKUDN010000000). The Comchoano-2 16S rRNA gene sequence is available via NCBI accession OM801197. The relative evolutionary distance (RED) is 0.83 to the closest relatives in GTDB release 95, as well as after re-calculation of all Comchoanobacterales identified in the present study. The phylogenetic position within the Comchoanobacterales order and the Comchoanobacteraceae family has been analyzed using both the reference genome (Extended Data Fig. 7b) and the 16S rRNA gene sequence (Fig. 2a). The GC content of the type genome is 41.6% and the genome length is 1.07 Mbp. The genome is high-quality, in two contigs, with standard measures (based on presence of single copy marker genes of Bacteria[17]) suggesting 96.55% completion and 0% contamination. However, this is likely an underestimate of the genome completion due the fact that no additional novelty was detected across multiple single cells. The type genome *Candidatus* Synchoanobacter obligatus sp. nov. originated from the North Pacific Ocean, from 20 m water depth. The genomes have limitations metabolically, apparently unable to synthesis fatty acids, vitamins, amino acids, or perform glycolysis, while encoding for a Type IV secretion system, pentose phosphate pathway, oxidative phosphorylation, a rhodopsin, and a putative ATP/ADP translocase.

Supplementary Figures

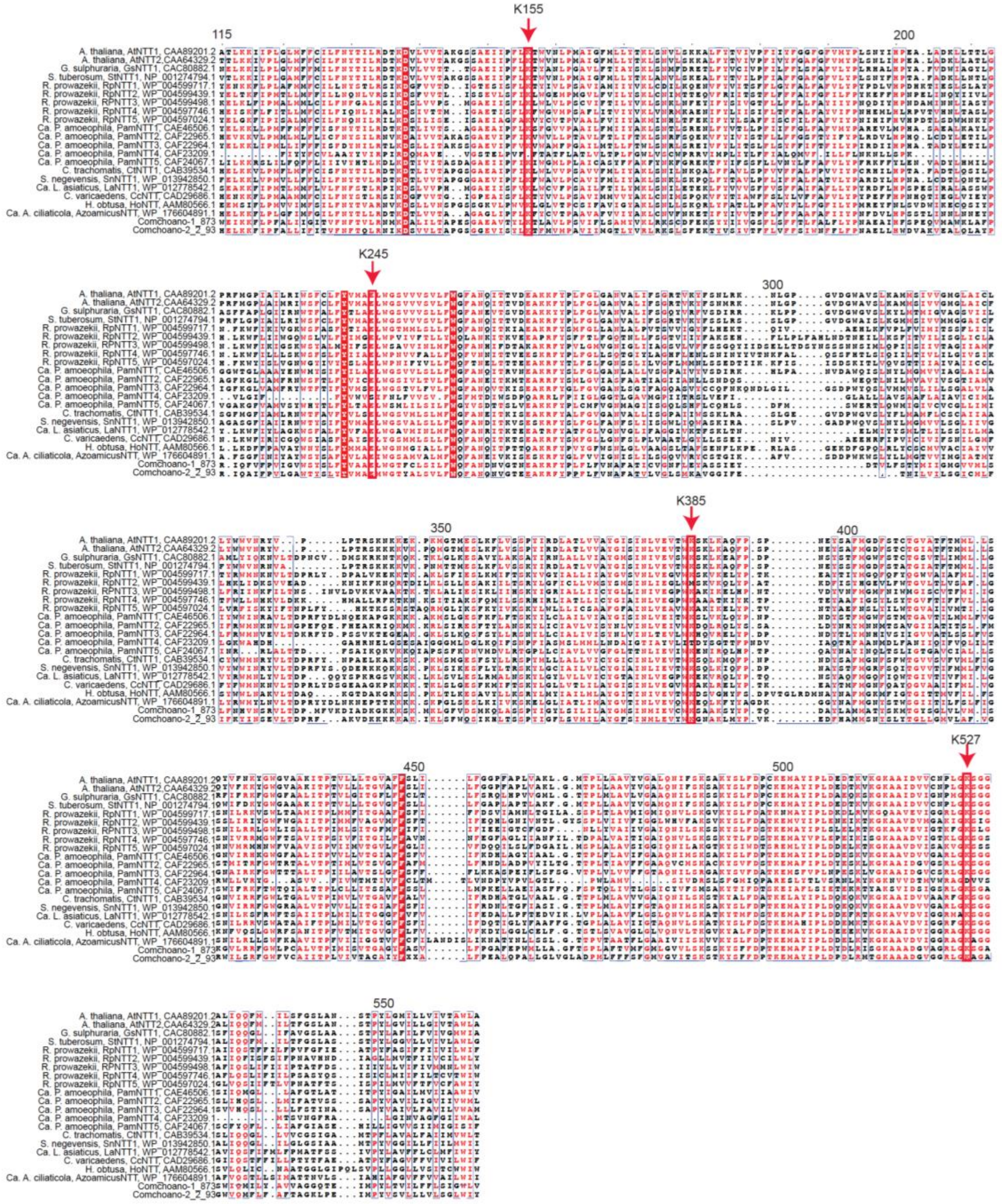

Supplementary Figure 1 | Conservation of motifs involved in nucleotide transport of Comchoano along with functionally characterized proteins. Letters in red are conserved across greater than 75% of the sequences for a given position, and those outlined in red are 100% conserved. The positions in blue are important for ATP/ADP translocation efficiency, substrate specificity, and counter exchange in *Arabidopsis thaliana*[42], as determined by site specific mutational analyses and are located in important transmembrane domains. The first 178 positions in the alignment (N-terminal end) which were highly variable were truncated for the

visualization (corresponding to 170 aa in CAC80882, *Galdieria sulphuraria* to 12 in CAF24067, *Protochlamydia amoebophila*). This alignment figure is modeled after an alignment of the same sequences (except Comchoano) reported in Graf et al. 2020.

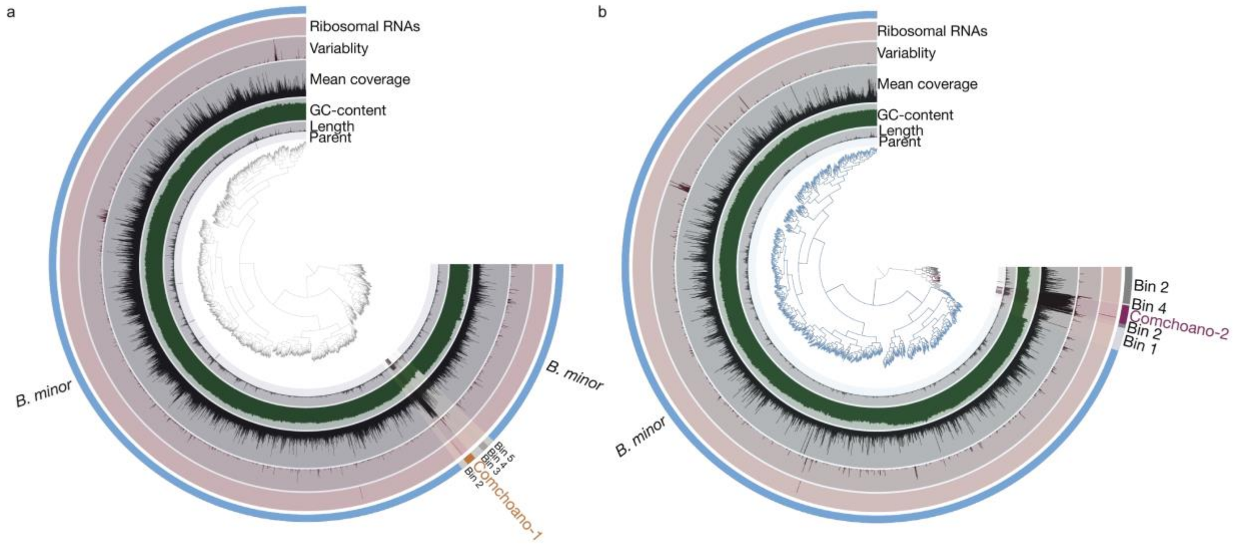

Supplementary Figure 2 | Genome binning of Comchoano-1 and Comchoano-2. **a,** Single cell sort of *B. minor* with Comchoano-1. Each column represents a single contig that was greater than 1 Kb in length. Contigs >20 Kb were artificially "split" for this analysis so that long contigs and short contigs are comparable for analysis. Split contigs were merged after binning. The inner dendrogram shows the clustering of the splits based on tetranucleotide frequency. The innermost row, Layer 1, shows splits that originate from a single contig; they are indicated here by adjacent ticks of identically colored gray scaled bars. Layer 2 indicates the length of each split. Layer 3 indicates the GC content of splits. Layer 4 indicates the average coverage of a given contig in the population sort. Layer 5 indicates the single nucleotide variability. Layer 6 indicates if a given contig encoded an rRNA gene sequence. Layer 7, the outermost layer, indicates the "bin" in which a given contig was sorted. **b,** Single cell sort of *B. minor* with Comchoano-2 with the same layers as in part **a.**
