# Supplementary material for: The microbiome of a bacterivorous marine choanoflagellate contains a resource-demanding obligate bacterial associate
Source: Nat Microbiol. 2022 Aug 15;7(9):1466–79. doi: 10.1038/s41564-022-01174-0 (PMC9418006; doi:10.1038/s41564-022-01174-0)
Supplement: Supplementary file 2 — Reporting Summary. [file 41564_2022_1174_MOESM2_ESM.pdf]

Corresponding author(s): David M. Needham, Alexandra Z. Worden

Last updated by author(s): May 16, 2022

Reporting Summary

Nature Research wishes to improve the reproducibility of the work that we publish. This form provides structure for consistency and transparency in reporting. For further information on Nature Research policies, see our Editorial Policies and the Editorial Policy Checklist.

Statistics

For all statistical analyses, confirm that the following items are present in the figure legend, table legend, main text, or Methods section.

n/a	Confirmed	
☐	☒	The exact sample size (*n*) for each experimental group/condition, given as a discrete number and unit of measurement
☐	☒	A statement on whether measurements were taken from distinct samples or whether the same sample was measured repeatedly
☐	☒	The statistical test(s) used AND whether they are one- or two-sided<br>*Only common tests should be described solely by name; describe more complex techniques in the Methods section.*
☒	☐	A description of all covariates tested
☒	☐	A description of any assumptions or corrections, such as tests of normality and adjustment for multiple comparisons
☐	☒	A full description of the statistical parameters including central tendency (e.g. means) or other basic estimates (e.g. regression coefficient) AND variation (e.g. standard deviation) or associated estimates of uncertainty (e.g. confidence intervals)
☐	☒	For null hypothesis testing, the test statistic (e.g. *F*, *t*, *r*) with confidence intervals, effect sizes, degrees of freedom and *P* value noted<br>*Give P values as exact values whenever suitable.*
☐	☒	For Bayesian analysis, information on the choice of priors and Markov chain Monte Carlo settings
☒	☐	For hierarchical and complex designs, identification of the appropriate level for tests and full reporting of outcomes
☐	☒	Estimates of effect sizes (e.g. Cohen's *d*, Pearson's *r*), indicating how they were calculated

Our web collection on statistics for biologists contains articles on many of the points above.

Software and code

Policy information about availability of computer code

Data collection	BD FACS Sortware (v1.0.0.650); Winlist (version 7.0, Verity Software House). MiSeq and HiSeq Reporter software as performed at the sequencing center that provided sequence data from our samples.
Data analysis	SPADES (v3.11.1); Bowtie 2 (version 2.3.0); blast 2.6.0; HTSeq-count (2.0.1); R v3.3.2; Prodigal (v2.6.3); tRNAscan-SE (v 1.3.1); eggNOG-mapper search (2.0.1); IQ-tree (v1.5.4); MAFFT (v7.222); trimAL (v1.2); AliView (v1.18.1); RAxML; FastTree (2.1.9); Cytoscape (v3.6.1); OriFinder (2008, webserver version); nucmer (v3); samtools (1.7); bcftools (v1); hmmscan (3.1b2), MacSyFinder (1.0.5), checkM (v1.0.13); MrBayes (3.2.6); GTDB-tk (1.4.0), QIIME2 (2018.8), dada2 (2018.8.0), epa-ng (v0.3.8), UCLUST (Edgar 2010), Cutadapt v.1.13,BD FACS Sortware (software v1.0.0.650), Geneious (20.2.4), diamond (v0.9.24.125), Predict Genome Auxotrophies tool (v.1.7.6), Pathway Tools (v22.0), txsscan (galaxy web-server version), GToTree (1.4.45), iqtree (2.0.3), iTol (version 5 and 6), cd-hit (4.8.1), and progressiveMauve (Version 2.4.0).

For manuscripts utilizing custom algorithms or software that are central to the research but not yet described in published literature, software must be made available to editors and reviewers. We strongly encourage code deposition in a community repository (e.g. GitHub). See the Nature Research guidelines for submitting code & software for further information.

Data

Policy information about availability of data

All manuscripts must include a data availability statement. This statement should provide the following information, where applicable:

- Accession codes, unique identifiers, or web links for publicly available datasets
- A list of figures that have associated raw data
- A description of any restrictions on data availability

Data availability Single and multi-cell sort raw data (short read archives, SRA) are available via NCBI Project Number PRJNA640955, which includes V4 16S rRNA gene

amplicon sequences from single cells, whole genome shotgun sequences from single cells, MBTS 16S and 18S V4 rRNA gene amplicons, and MBTS V4-V5 rRNA gene amplicons (see Supplementary Data 12 for individual list of SRA accessions). 18S V4 rRNA gene amplicons are available as part of Needham et al. 201918. Comchoano-1 and Comchoano-2 whole genome sequences are available via accessions CP092900 and JAKUDN000000000 and their full-length 16S rRNA gene sequences are deposited as OM801198 and OM801197. Alignments, tree files, and processed amplicon data are available via FigShare (doi: 10.6084/m9.figshare.c.5850662).

Field-specific reporting

Please select the one below that is the best fit for your research. If you are not sure, read the appropriate sections before making your selection.

☐ Life sciences ☐ Behavioural & social sciences ☒ Ecological, evolutionary & environmental sciences

For a reference copy of the document with all sections, see nature.com/documents/nr-reporting-summary-flat.pdf

Ecological, evolutionary & environmental sciences study design

All studies must disclose on these points even when the disclosure is negative.

Study description	Single cells were sorted from seawater and genome sequenced. There was a total of 188 cells sorted from this experiment all of which had 18S and 16S PCR performed on them. We report the ASV data for all of those that produced results. We found that 1% of the choanoflagellate cells had one of two types of Comchoano bacteria associated with them which we report here. We also contextualize these findings by demonstrating the Comchoano and choanoflagellate abundance in available datasets such as from the location of study the Monterey Bay, as well as a survey that also had 18S and 16S data from the San Pedro Ocean Time-series. We also surveyed ASV data from global circumnavigations to demonstate the abundance of Comchoano and choanoflagellates in the global ocean. Most of the environmental ASV data were not in replicate technically (whether they are original to our manuscript or the other ASV datasets) but were in either time-series or spatial distributions allowing a sense of their variability over space and time. Otherwise, the technical sample design structures of e.g. factorial, nested, hierarchical per se were not systematically followed in our environmental study.
Research sample	Seawater from offshore central California, USA, see below within "Data Collection". The sample is chosen to be basically representative of the offshore central California coast during spring and coincided with sampling opportunity. Amplicon sequencing from bulk samples and metatranscriptomes are from seawater filtered via vacuum pump, with no further manipulation. For the flow cytometry, lysotracker staining as added to help identify heterotrophic protists by staining of their acidic components (such as food vacuoles). The cells sorted of B. minor are expected to be representative of the choanoflagellate community on the day of sorting in the surrounding waters. Malaspina, Tara, and San Pedro Ocean time-series data were downloaded from previously published datasets as described in the manuscript. Amplicon sequencing and metatranscriptome sequencing were all sequenced on Illumina sequencing and deposited as raw data via NCBI Project Number PRJNA640955.
Sampling strategy	NA, no sample size calculation was necessary. There are no statistical analyses of populations etc.
Data collection	Seawater for sorting was collected on 20 March 2014 at Station M2 (36.688°N, 122.386°W, Fig. 1a) using Niskin bottles mounted on a CTD rosette. Water from 20 m depth was pre-filtered through a 30 µm mesh, concentrated by gravity over a 0.8 µm Supor filter to about 250 times concentration, stained with LysoTracker Green DND-26 (final concentration, 25 nM), and run on a BD Influx flow cytometer equipped with a 488 nm laser using sterile nuclease-free 1x PBS as sheath fluid. The sorted population was discriminated based on positive LysoTracker signal (i.e., fluorescence detected in 520 ± 35 nm bandpass filter under 488 nm excitation) as compared to an unstained sample and absence of chlorophyll-a autofluorescence (i.e., 692 ± 40 nm filter), and similar Forward Angle Light Scatter (FALS) to select coherent populations of heterotrophic eukaryotes (Fig. 1b). Single cells were sorted into a 384-well plate using the Single-Cell sorting mode from the BD FACS Sortware (software v1.0.0.650). A subset of wells was left empty or received 20 cells for negative and positive controls, respectively. The plate was illuminated with UV for 2 min prior to performing the sort and covered with foil and frozen at -80°C immediately after sort completion. Camille Poirier performed and recorded all flow cytometric analyses. David Needham recorded all genome sequencing data and choanoflagellate single cell amplicon data. Charmaine Yung processed amplicon gene surveys from MBTS. Lisa Sudek performed PCR on MBTS samples. AJ Limardo helped with MBTS sampling.
Timing and spatial scale	In addition to the flow cytometric single cell sorting sampling described above, seawater samples were taken for rRNA gene surveys and metatranscriptomic surveys from a total of 3 months in 2014 and one in 2015 from surface waters inside and outside of the Monterey Bay. Other months sampled were not analyzed for this project due to timing and applicability to the manuscript (overlapping with single cell sorting most closely so provide the most relevent context). Additionally, previously published data were used from the southern california oceanographic region's San Pedro<br>Time-series (2010) as well as surface waters from the circumnavigating Malaspina and Tara Ocean's cruises were used.
Data exclusions	None
Reproducibility	188 single choanoflagellate cells were investigated with 12% of them found to be associated with 1 of 2 types of Comchoano. Multiple nearly identical Comchoano genomes were recovered from single cell sequencing of choanoflagellate cells. Extensive analyses were performed to demonstrate the level of single nucleotide variation between the genomes. All the single cell analyses report come from a single experiment with B. minor never becoming prevalent enough to repeat the experiment, though we do find that B. minor and Comchoano and their relatives globally distributed
Randomization	not applicable, samples were sampled from diverse, discrete microbial community where randominzation is not relevent, no human subjects

Blinding | not applicable, no human subjects were used.

Did the study involve field work? ☒ Yes ☐ No

Field work, collection and transport

Field conditions: The ocean sample on which sorting was performed was collected from 20 m depth in the mixed layer at station M2 (36.688 °N; 122.386 °W, 56 km from shore, Fig. 1a) on 20 March 2014 where temperatures were 12.7 at the depth of sampling, 12.8 at the surface and above 12 to the thermocline around 45 m. The 20m depth sample was the chlorophyll maximum depth where the chlorophyll concentration was 2.1 µg/L (1.0 µg/L at the surface and 1.8 µg/L at 40m)

Location: main flow cytometric single cell sorting undertaken at 20 m depth: 20 March 2014 in the northern pacific ocean (36.688°N, 122.386° W). Like this sample, the others are presented with their latitude and longitude in our manuscript (or in previously published reports, their original publication).

Access & import/export: Sampling was from surface ocean waters in the US economic zone or international waters and processed primarily by scientists and facilities in the US, since moved to the EU (Germany). The samples were water samples of typically 10 L from various depth in the water column, especially the surface photic zone and were as such non invasive, do not harm the environment and did not involve CITES species or any other endangered or animals of any economic value.

Disturbance: a maximum of 30 L of ocean water was collected for a given sample, this does not disturb the ecosystem.

Reporting for specific materials, systems and methods

We require information from authors about some types of materials, experimental systems and methods used in many studies. Here, indicate whether each material, system or method listed is relevant to your study. If you are not sure if a list item applies to your research, read the appropriate section before selecting a response.

Materials & experimental systems

n/a	Involved in the study
☒	☐ Antibodies
☒	☐ Eukaryotic cell lines
☒	☐ Palaeontology and archaeology
☒	☐ Animals and other organisms
☒	☐ Human research participants
☒	☐ Clinical data
☒	☐ Dual use research of concern

Methods

n/a	Involved in the study
☒	☐ ChIP-seq
☐	☒ Flow cytometry
☒	☐ MRI-based neuroimaging

Flow Cytometry

Plots

Confirm that:

☐ The axis labels state the marker and fluorochrome used (e.g. CD4-FITC).

☐ The axis scales are clearly visible. Include numbers along axes only for bottom left plot of group (a 'group' is an analysis of identical markers).

☐ All plots are contour plots with outliers or pseudocolor plots.

☐ A numerical value for number of cells or percentage (with statistics) is provided.

Methodology

Sample preparation: Sorted seawater was collected on 20 March 2014 at Station M2 (36.688°N, 122.386°W, Fig. 1B) using Niskin bottles mounted on a CTD rosette. Water from 20 m depth was pre-filtered through a 30 µm mesh, concentrated by gravity over a 0.8 µm Supor filter to about 250 times concentration and stained with LysoTracker Green DND-26 (final concentration, 25 nM).

Instrument: BD Influx flow cytometer equipped with a 488 nm laser using sterile nuclease-free 1x PBS as sheath fluid.

Software: BD FACS(TM) Software v 1.2.0.142 (run software); Verity Software House WinList 9.0 (figure display software)

Cell population abundance: There is no population abundance analysis in this manuscript. The sorting was used to separate cells into individual wells that were then sequenced (as described in methods). The pre-concentration methods used preclude derivation of numerical information for the flow cytometric analyses. Cell population abundances were mostly inferred from rRNA gene sequencing locally and at locations around the global ocean.

Gating strategy: The sorted population was discriminated based on positive LysoTracker signal (i.e., fluorescence detected in 520 / 35 nm

Gating strategy

bandpass filter under 488 nm excitation) as compared to an unstained sample and absence of chlorophyll-a autofluorescence (i.e., 692 / 40 nm filter), and similar Forward Angle Light Scatter (FALS) to select coherent populations of heterotrophic eukaryotes (Extended Data Fig. 1a). Single cells were sorted into a 384-well plate using the Single-Cell sorting mode from the BD FACS Sortware (software v1.0.0.650).

☒ Tick this box to confirm that a figure exemplifying the gating strategy is provided in the Supplementary Information.
